# Book Reviews

**Published:** 1820-04

**Authors:** 


### CRITICAL ANALYSIS

OF

### RECENT PUBLICATIONS, IN THE DIFFERENT BRANCHES OF MEDICINE AND SURGERY;

### SELECT MEMOIRS, AND HISTORIES OF CASES;

### In the Literature of Foreign Nations.

---

Πατρίδες ἄρα
Ἄνδρασιν, οὐ πατραῖς ἄνδρες, ἀγαλλόμεθα.

---

***Medico-Chirurgical Annuary of the Civil Hospitals and Infirmaries of Paris; or, a Collection of Memoirs and Observations by the Physicians and Surgeons of those Establishments.*** **4to. pp. 636; with fourteen Plates in Atlantic folio. Paris, 1819.**

[In continuation from page 240.]

***Observations serving as Materials for a Memoir on Apoplexy.***
By M. Lerminier, Physician to the Hôtel-Dieu.

M. LERMINIER relates here, nine cases illustrative of the process by which blood extravasated in the brain becomes enveloped in a cyst, and at length removed by *absorption*. As we discern nothing of important novelty in these observations,* in this point of view, we shall pass them over solely with the remark, that they tend to confirm the establishment of the notions first clearly advanced on this subject by Riobe.

M. Lerminier draws no inferences from these cases on the present occasion, but he states that he shall do so in another memoir. We shall make a few remarks on them in relation to therapeutics. Several of the patients here alluded to, recovered from the apoplectic affection which, it appears, accompanied the extravasation of blood in the first instance, and afterwards died with symptoms of inflammation of the brain, evident during life; and dissection showed that effusion of serum and coagulable lymph had taken place, subsequently, it appears, to that of the blood. These signs of inflammation extended from the parts surrounding the coagulum, and probably arose from the irritation caused by this, as a foreign body, and were, we suppose, only the consequences of a too intense degree of that salutary process by which the cyst above designated is formed around the coagulum. We think it important, that the disposition to such an occurrence, after recovery from apoplexy, should be borne in mind; and that, had some of M. Lerminier's patients been bled, and submitted to other analogous and appropriate measures, at the onset of the latter series of symptoms, they would very likely have been preserved.

---

* An account of this point of pathology was given in the Historical Sketch of the Progress of Medicine, inserted in this Journal for January 1819.

As the subject of apoplexy is now before us, we pass over some observations by M. Husson, for the present, to the following memoir.

*A new Division of Apoplexies.* By A. Serres, Chevalier of the Legion of Honour, one of the Physicians of the Hospital la Pitié, Superintendant of the Anatomical Investigations at the Hospitals, &c.

Impressed with a sense of the vague and unsatisfactory nature of the prevalent doctrines on apoplexy, and of the advantages that would accrue to the practice of medicine from more precise knowledge of this point of pathology, M. Serres, for seven years previously to the time at which he composed this memoir (in the year 1814), engaged in the investigation of this subject with particular assiduity; and the results of his researches in the wards of hospitals, dissections of one hundred bodies of patients of this affection, and several experiments on animals, have led him to form some new opinions respecting the origin of apoplectic maladies, and especially to combat the doctrine, that pressure of the brain is the cause of the phenomena by which they are characterized. We shall first examine the experiments he instituted on several animals, with the object of ascertaining the truth or falsity of the opinion last mentioned.

He trepanned an old dog on the middle part of the cranium, corresponding to the superior longitudinal sinus; thrust a fine bistoury completely through the superior and inferior parietes of this sinus, and closed the external opening, in order that the effusion of blood might take place within the cranium. The dog was let loose: he ran about the room, trying to make his escape. Three hours afterwards, his natural appearance and actions were so little altered, that it was doubted whether effusion of blood had occurred. The cranium was opened: a very voluminous coagulum of blood was found in the fissure between the lobes of the brain, and a second, smaller, on the left hemisphere. Similar experiments were made on a young dog, rabbits, and several species of birds, with similar results. Neither somnolency, nor other symptoms of apoplexy, were observed. Here, M. Serres says, he reduced the question to its most simple form: he did not interest the substance of the brain; any symptoms that might have arisen must have been attributed to pressure on this organ; but none thence arose. A strong presumption is then furnished, he concludes, against the opinion generally received; because the effusion which here took place might be compared to those which occur between the dura mater and the arachnoid membrane, or between the latter and the pia mater, as a consequence of apoplexies;—apoplexies in which the cerebral substance is free from lesion.

We think that M. Serres has here drawn inferences which are not so well founded as he seems to imagine. We submit our reasons for this opinion, not with confidence, because they are not, in every point, inductions from actual observations, but as the results of what we think plausible rational arguments.

Every person acquainted with the principles of hydraulics must perceive, on reflecting on the construction of the brain and cranium, that if the skull is at any period of life filled with the brain, it must for ever afterwards be filled either with it, or with this organ and certain fluids, whilst the periphery of the skull remains entire. A vacuum cannot exist between the brain and cranium. Now, we know that the brain, alone, or with some fluids extraneous to that organ, does fill the cranium in the natural state of these parts : it follows hence, that, when preternatural effusion of blood or any other fluid takes place in it, either a similar quantity, in respect to volume, of the brain must be removed, or a similar diminution of the fluids contained in its vessels must take place. Let us now suppose that a blood-vessel of a certain diameter bursts in the brain : it is obvious, that the blood flowing out from this ruptured vessel will receive from the heart such a degree of momentum as will cause it to compress, to a certain extent, such of the surrounding vessels as are naturally kept expanded by a force less than the compressive force; and the cavities of some vessels may thus be quite obliterated. Those portions of the brain, then, that receive their blood from those obliterated channels, will have their functions suspended for want of blood, when anastomosing vessels are not so disposed as to supply the deficiency; the consequences of which will be cessation of those functions of the rest of the body that depend on the influence of those portions of the brain: and hence, according to the degree, extent, and situation, of the compression, there will be stupor, paralysis, apoplexy, &c. But, when fluid is effused from extremely small vessels, its compressive power can be relatively but slight on any given portions of the surrounding brain, whilst the natural actions of such portions continue: its compressive force will be widely diffused, and will not wholly obliterate any vessels, the expansive force of whose contents, by their gravity and momentum, is superior to the compressive power of the effused fluid. Hence a considerable quantity of serous fluid is sometimes effused within the cranium, and yet the functions of no portion of the brain are apparently suppressed. We mention this now, as an answer to an objection that might be adduced against the foregoing notions. We shall recur to this point hereafter.

Let us now consider the state of the brain and cranium in the experiments of M. Serres. He removed a portion of the periphery of the skull, and thrust an instrument through the meninges of the brain. We have repeatedly found, in making experiments on living animals, that the brain, on being exposed to the atmosphere in this way, shrunk, and really did not fill the cranium. We do not pretend to explain the reason of this: it may have been from the influence of relative coldness, or other chemical qualities of the air, on the brain ; but we assert the fact. Here, then, there is space between the cranium and the brain for blood to be effused in, without producing compression of the brain; not taking into account that gained by the removal of a portion of the skull, and the little resistance of the integuments of the head compared to that made by the bone, in the experiments of M. Serres. We must also bear in mind, that, in these experiments of M. Serres, the blood was effused from a venous sinus, not from an artery, and therefore that it was poured out without much momentum; —a circumstance of great importance in regard to its immediate agency as a compressive power. This diminution of the volume of the brain, on this organ being thus exposed to the air, may, in one respect, be compared to that produced by the slow and gradual accumulation of serous fluids in the cranium. No one part undergoes such great compression as to render it unfit for performing its functions.

The foregoing observations are, we think, calculated to show that the inferences of M. Serres are not so rigorously deduced as he imagines; and then we have positive evidence that pressure on the brain will produce the phenomena of apoplexy, in cases of depression of the skull, the effects of pressure on spina bifida and tumors of the head of an analogous nature in infants, wilful or accidental pressure on the heads of persons who have been trephined, &c.

M. Serres then made some experiments with the view of ascertaining the effect of effusion of blood in the ventricles. He trepanned an adult dog over the middle of the superior longitudinal sinus; he passed a very narrow bistoury in the sinus between the hemispheres of the brain as far as the corpus callosum, then turned the point of the instrument to the left, and thrust it into the ventricle of this side. The bistoury was withdrawn, and the external opening closed. The animal seemed to have a sort of vertigo, which continued for a minute: he was dull during the rest of the day, had a little disturbance of the pulse, and suffered much change in his appearance; but there were no signs of somnolency. He passed the night in a state of agitation. In the morning, he walked about the room, and appeared less affected than on the preceding evening. On opening his cranium, the effused blood had filled* the fissure between the two hemispheres, penetrated the left ventricle, which contained an ounce and a half of it; and a little collection had formed in the anterior part of the corpus callosum.

A rabbit two months old was submitted to the same experiment. On being instantly let loose, he ran about the garden for five or six minutes without stopping; he was then caught, his cranium opened, and effusion was found to have taken place: the fluid "occupied the same fissure (between the hemispheres), the interior of the right ventricle (which was here opened), and the base of the cranium, where it arrived by means of a little loss of substance at the anterior part of the corpus callosum."

As far as *pressure* of the brain is concerned, our former remarks seem to be applicable to these cases.

M. Serres remarks on these experiments: "The cerebral substance not having been interested, or but slightly interested in part of the corpus callosum, it might be believed that the brain had not been submitted to the immediate action of the weight of the blood. To obviate this objection, and to place the animals in the condition in which apoplectics are so frequently found, it became necessary to hollow out artificial excavations in the brain itself, and to ascertain the result."

---

* This is the expression of M. Serres: but there is no vacancy to fill, in the natural state of the parts.

With this view, he trepanned an old dog on the side of the posterior part of the longitudinal sinus, thrust a bistoury into the left hemisphere, and removed a portion of it (two drachms.) No somnolency, no oppression of respiration. A cavity containing a coagulum of the size of a nut, was found in the middle part of the hemisphere. In another animal he made a cavity in both hemispheres: the same result; no apoplectic symptoms. The same result in a pigeon, in which both the hemispheres were pierced with a very large pin.

An artificial cavity was hollowed out of the middle part of the hemisphere of a dog, by removing a certain quantity of the brain: a piece of cork of the figure of a plug was thrust into it, "in order to increase the compression." There was complete hemiplegia, but neither apoplexy nor somnolency.

M. Serres draws the following conclusions from the above-mentioned experiments;

"Thus, effusions of blood do not produce apoplexy, whether they are situate between the cranium and the dura mater, between this membrane and the brain, or in the great fissure between the hemispheres resting on the corpus callosum; whether a cavity be found in the anterior, middle, or posterior, part of the hemispheres, or these parts transpierced throughout; or, lastly, when they traverse the corpus callosum, and penetrate into the ventricles, and fill these cavities. The same results in birds, in rabbits, and in dogs. Apoplexy in man, then, cannot be attributed to the presence of effused blood, whatever part it occupies, whether it be without the brain, or in the cavities of this organ, or lodged in its proper substance."

We cannot join in this conclusion, for the reasons we have already advanced; and because we think that experiments on the brain of animals so low in the scale, in respect to the development of this organ, in comparison to man, are not so strictly applicable to him as M. Serres infers. We proceed to the examination of the arguments by which M. Serres supports his propositions.

He first adduces several cases in which either serum or blood was found in the cranial cavity, in some instances in the substance of the brain, after death, when no symptoms of apoplexy had been present; and others where, it appears, persons had recovered from the immediate symptoms of apoplexy, although a coagulum of blood of considerable size existed in the brain. But these are not positive arguments in support of his proposition, that "*les epanchemens sanguins ne produisent point l'apoplexie.*" The facts in question only show that sanguineous effusion *may* take place without producing apoplexy. Another argument advanced by M. Serres is, "If the compression by the fluids were the cause of apoplexy, a rigorous consequence is this: *no apoplexy without effusion.*" Now this, we consider, is not a necessary consequence, because *compression* may be produced by other causes than effused fluids; and, though effected by diverse circumstances, its influence on the functions of the brain may be identical. We cannot but be pleased to find any thing like *logic* adduced in physiology, but the logic of physiology (we must not be censured for this *tautology*, the common use of the word physiology obliges us to use it) is not that of dynamics. Certain causes do not here always produce their general, and we may say, *proper*, effects; and identical effects (as far as our senses inform us) often result from, or follow, diverse causes. This last proposition will not appear so objectionable as it might on a first view, if it be considered that these diverse causes may have relations to the existence of the functions of the brain that are common to them all, and that it is through those relations that the effects in question are produced.* We think, then, the experiments on animals of M. Serres prove nothing of what he argues for; and that his observations on dead human bodies only show, that fluids may be effused in the cranial cavity without producing apoplexy, and that apoplexy may exist without effusion of fluids.

M. Serres says, "How can we suppose that, in sudden attacks, a sudden effusion takes place without any precursory symptoms, an instantaneous rupture of an artery, or a shower of serum, if such an expression may be permitted?"

"After having shown in what false courses the inclination to systematize had led observers," says M. Serres; "after having shown that effusions are the effect, and not the cause, of apoplexy, as was supposed; after having pointed out the errors inherent in all the divisions hitherto proposed, it remains for me to develop those which observation of the phenomena of apoplexies, and dissection of dead bodies, have given rise to, and confirmed, for several years."

We must first remark, that it is curious that M. Serres should have neglected to notice the observations of Dr. Cheyne, and some other physicians, who long since showed what he here chiefly argues for; and now we will expose to our readers the new division of M. Serres.

On assiduously studying the origin and development of apoplexies, and comparing the symptoms which they presented, M. Serres says, "he soon perceived that they assumed two different forms: the one, simple and without complication with paralysis; the other always complicated with loss of motion of one side." What are these propositions which M. Serres *ne fut long temps à appercevoir*, when stripped of the affected *systematic* pomp with which he advances them, but the common observation, that paralysis sometimes accompanies apoplexy?

"But is this remarkable circumstance," he continues, "the effect of chance, or does it depend on some cause recognizable in the dead body? This is the answer with which histories of a hundred apoplexies have supplied me. Of this number, twenty-one were simple, seventy-nine presented complications with palsy; of the first, dissection furnished the following results: 1°, sixteen with serous effusion, either both in the ventricles and convolutions of the brain, or in the ventricles and those convolutions distinctly and solely; 2°, one with sero-sanguineous effusion in the left ventricle; 3°, two with a similar effusion

---

* How many errors have arisen in pathology from the principles of *causation* being so imperfectly understood by the generality of us! If our judgment be correct, no trifling benefit will result in this, as well as in a multitude of other instances, from the influence of the very extraordinary work of Mr. Pring, *the General Indications which relate to the Laws of the Organic Life.*

between the arachnoid and the pia mater, on both the hemispheres; 4°, two presented no effusion.

"In all, the brain was healthy; the meninges were altered in different degrees: 1, in those in which the duration of the disease had been long, and when the serous effusion was abundant, the pia mater was injected, the vessels very much dilated, the arachnoid opaque and thickened; 2, in that where the ventricle alone had been the seat of a sero-sanguineous effusion, the arachnoid, slightly opake in the rest of its extent, was reddish in the interior of this ventricle, studded with very numerous miliary granulations, confined like the effusion; 3, the arachnoid over the hemispheres was evidently inflamed in the two which presented a similar effusion on the hemispheres; 4, the arachnoid presented a dryness, with thickening and membraniform productions in those where there was no effusion. Does not this constant relation between the meninges and the effusions indicate that these two effects were allied one to the other? Do we not see in the different alterations of the arachnoid and the pia mater the cause of the different effusions, and of the absence of all exhalation in the two last cases?"

We must interrupt the author's course here, just to remark, that the absence of effused fluid after death is not a proof that it had not existed in any stage of the disease; on the contrary, the state of the serous membranes described by M. Serres is found frequently to have been preceded by increased serous exhalation. That is, we find in inflammation of serous membranes situate in parts more under our immediate inspection, that increased secretion of serum often precedes, as above indicated, such a stage of inflammation as is productive of *membraniform productions.* The serous fluid is sometimes afterwards absorbed, whilst the coagulated lymph remains. Thus, it appears that, in the hundred cases mentioned by M. Serres, certainly all but two, and probably the whole, were accompanied with effusion of fluids; for, as the reader will presently find, all of the *complicated* cases presented signs of it in the dead body. We must leave the reader to make his own reflections on these facts: this article would acquire far too great an extent, if we were to stop to adduce all, even the most immediate, inferences to which the above observations may give rise.

M. Serres continues, "May not this irritation of the arachnoid of the ventricle, and of the portion which covered the hemispheres, be considered as having given rise to the sero-sanguineous effusion? A multitude of facts which I have since observed, permit me to reply in the affirmative to these questions; and I believe I may establish as a principle, that, in this species of apoplexy, the meninges are principally and primitively affected, and that the different effusions which may be found are only the effects of their alteration."

We also reply in the affirmative to the latter question with almost absolute confidence; but we do not see how the acknowledgment of the relations here designated favours the author's doctrine, that compression of the brain does not produce *apoplexy.*"

"The above characters," M. Serres proceeds to state, "placed in opposition with the examination of the bodies of persons dying after having apoplexy complicated with paralysis, acquire a higher degree of certitude. Here there were no serous orsero-sanguineous effusions in the natural cavities of the brain, or in the space between its duplicatures; the malady was in a manner foreign to them; but the brain, materially altered in its structure, appeared to have exclusively the centre of activity of the apoplexy. Cavities were hollowed in its structure, and in different parts of the substance of its lobes; the cerebral laminæ all around them were irritated, reddish, yellowish, and hardened, according to the duration of the interval which elapsed from the formation of the cavity to the time of death. The blood which filled these excavations, or these caverns, according to the expressions of Wepfer and of Morgagni, was either coagulated or semi-fluid, according as the cavities were situate in the cortical or the medullary substance, the corpora striata, the thalami optici, or even the interior of the ventricles, where it had often penetrated after having broken down their parietes. There was no doubt respecting the manner in which this was effected; for a fine injection, forced into the carotids, penetrated into the cavern, and simulated in a manner the effusion.

"Let me be permitted, before I pass further, to advance the two following questions:

"Why had all these last apoplexies been complicated with paralysis?

"Why did not the first present, during their progress, any lesion of the power of motion? May we not answer that, in the complicated apoplexies, the paralysis was a necessary effect of the organic alteration of the brain; whilst, in the simple apoplexies, the brain being healthy, the power of motion should also preserve its integrity?

"If these be true, may we not form the three following propositions?

"1. When an apoplexy does not present in its course any trace of paralysis, may it not be presumed that it has its seat in the meninges, and that the brain is not the centre of the activity of the disease?

"2. When, on the contrary, paralysis becomes complicated with apoplexy, it is not the meninges, but the brain, that is principally the seat of the irritation.

"3. Serous, sero-sanguineous, bloody, or purulent effusions, are the effects of irritation of the meninges, of the brain, or of ruptures of the arteries or veins, which may happen in the course of apoplexies.

"If I have well interpreted the facts, it appears to me, that it is easy to assign to those apoplexies the denominations which are proper for them. I propose to designate the apoplexies without paralysis by the term *meningeal apoplexies*, and those which are complicated with paralysis, by that of *cerebral apoplexies*. I shall treat in another memoir of apoplexies of the *cerebellum*."

We shall take these propositions into consideration in another article, as well as the observations intended to illustrate them, which M. Serres has adduced.

### *On Mediate Auscultation; or a Treatise on the Diagnosis of the Diseases of the Heart and Lungs, principally founded on this new Mean for Exploration.* By R. T. H. Laennec, M.D. &c. &c. Vol. II. Paris, 1819.

[In continuation from page 249.]

The next subject treated of in this work, is *emphysema of the lungs.* This, in a slight degree, is not an uncommon affection, and has been noticed by Dr. Baillie, and several former authors, but not so accurately described as it is here by M. Laennec. It consists, in its less intense degree, in an irregular dilatation of the air-cells of the lungs, forming cavities varying in size from that of a millet-seed to that of a French bean; they do not ordinarily project from the surface of the lungs, though they sometimes form slight spherical prominences. In the latter case, the lung appears to be vesicular, like that of the *batracians* amongst reptiles. The disease may exist in this state, and yet the air be contained only in the proper air-cells; but, when the distension becomes greater, the cells burst, and effusion of air takes place into the cellular texture, giving rise to phlyctenæ more or less voluminous: sometimes they acquire the size of an egg, but readily subside under moderate pressure by the hand. These cavities sometimes contain a very small quantity of blood. This affection, though but very rarely, has been found joined with the dilatation of the bronchial tubes before described. The lung is necessarily increased in bulk when only one is affected, the healthy one giving way to the pressure of that which is diseased; and, when both are affected, they are found to spring out and expand with considerable violence, when removed from the cavity of the chest. They are less compressible and firmer than in the healthy state; but their specific gravity is diminished, and they are more dry than ordinarily. No sanguineous or serous engorgement of the organ is generally present; but this complication has been witnessed.

The general signs of this disease are very equivocal: dyspnœa constitutes its principal character; and, during life, it has been considered as *asthma.* The difficulty of respiration is habitual; but it is increased by most of the causes which aggravate dyspnœa in general. There is no fever, and the pulse is commonly regular. The colour of the skin, and the appearance of the body generally, present nothing peculiar when the affection is but slight in degree; but, when it is considerable, the skin is ordinarily of a dull, and as it were dirty, aspect, with a violet tinge in some parts. The lips are of a violet hue, and appear tumefied. All the patients observed by M. Laennec had an habitual cough, sometimes coming but seldom, slight, and dry, or followed with a little greyish, clear, and very viscous, mucus; sometimes severe, coming in violent fits, and accompanied with copious expectoration of thick mucus. The disease often commences in infancy; it may continue a great many years, and the patient may even live to an advanced age.

The habitual, and often very great, effort required to dilate the lungs, has frequently produced at length hypertrophy or dilatation of the heart.

On applying the stethoscope to the parietes of the chest in this disease, respiration is not heard in the greater part of this cavity, although it renders a very clear sound by manual percussion; and the sound of respiration is very weak, even where it can be perceived. From time to time, and during a very deep inspiration, slight hissing *rattles*, analogous to the sound of a little pump-sucker, may be heard in the points corresponding to the affected part, or generally over the chest when the affection occupies the whole of the lungs.

This absence of the sound of respiration in a subject whose chest is sonorous on percussion, will enable us to distinguish emphysema of the lungs from all other affections, excepting pulmonary catarrh, and *pneumo-thorax*, or effusion of air into the cavity of the pleura; and the former affection may be confounded with partial emphysema: but attention to the other attendant signs, will enable every accurate observer to distinguish them. When emphysema of the lungs has long existed, the parietes of the chest become extended, more vaulted in figure, and the intercostal spaces are larger than natural. M. Laennec relates several cases of this disease, with necroscopic observations, which furnish the basis for the above general remarks.

M. Laennec next treats of *accidental productions developed in the lungs:* those he notices are, 1, cysts, properly so termed; 2, cysts containing vesicular worms; 3, fibrous, cartilaginous, osseous, and osseo-cretaceous masses; 4, tubercles; 5, a species of morbid production which he calls *encephaloid matter;* 6. melanosis.

By the term *cyst*, the author intends to signify, with the greater part of modern pathologists, an accidental membrane forming a sort of sac without an opening, ordinarily of an oblong-spheroid shape, sometimes however irregular and unfructuous, containing a liquid or semi-liquid matter, secreted by the membrane which forms the cyst. There is another species of cyst which envelopes more solid substances and bodies foreign to the animal economy, as tuberculous matter, the different species of cancer, &c. but it is only of the former that M. Laennec here treats.

These cysts are always formed by a natural tissue; that is to say, a tissue similar to some one of those which naturally exist in the body in the state of health. In the greater number of instances, the membrane which constitutes them resembles either the serous or mucous membranes. Sometimes a layer of fibrous tissue, or of condensed cellular membrane, envelops the cyst exteriorly, and unites it to the neighbouring parts. Sometimes cysts are found composed wholly of a mixture of the two latter tissues, to which the cartilaginous tissue then generally becomes joined, and even more or less voluminous osseous laminæ. The internal surface of the latter cysts hardly ever presents the smooth and polished aspect of those formed by serous and mucous membranes.

Cysts of this species are, of all accidental productions, those which are most rarely developed in the lungs of man.

The only species of *vesicular worm* which M. Laennec has found in the lungs appertains to the genus which he terms *acephalocystes*: these are what are commonly known by the name of *hydatids*.

M. Laennec believes the *hydatid* to be an *animal*; and he says he has seen all the degrees of their reproductive process, which takes place, as is the case with certain polypi, by a sort of bud, which first appears in the substance of the paries of the worm, protrudes from one or other of its surfaces, becomes hollow, takes a spherical form as it enlarges, and terminates by becoming detached from the parent animal.

The proper hydatid is always enclosed in a cyst, which separates it from the surrounding parts. These cysts are ordinarily fibrous; but points of cartilaginous or of osseous texture, are sometimes also found in them. The internal surface is but rarely smooth; sometimes it is covered by opake, semi-concrete albuminous matter, of the colour of dull yellow-ochre. Hydatids are often found in the human lungs.

Cartilaginous, osseous, cretaceous, and analogous concretions, are also often seen in these organs, and have been accurately described by several former authors.

The *encephaloid* or *cerebriform matter*, is regarded by M. Laennec as a species of *cancer*; and it is the only kind, he says, that he has found in the lungs. It exists under three different forms: that is, encysted, collected into irregular masses; and without a cyst, or infiltrated in the pulmonary tissue. In whatever form it exists, it presents three different periods of its development: that of its formation, or state of *crudity;* that of its full development, in which it especially resembles the cerebral substance in its appearance; and that in which it has become softened.

During the greater part of the existence of these tumors, there is no evident fever; and, in many cases, death occurs without the pulse having ever presented any remarkable alteration. When febrile symptoms take place, they appear to be owing to accidental circumstances; that is, when they, by their position, oppress important parts, or occasion more or less extensive inflammation in the surrounding structure. They may exist for a long time without producing remarkable emaciation of the body generally. But this symptom is constant towards the epoch of the termination of the disease, and it then proceeds in a very rapid manner. The only cases where death takes place without it, are those in which this is caused by the situation of the tumors, and by the pressure they exert on important organs, as the brain or the lungs.

The productions called *melanoses* by M. Laennec, are of a consistence equal to that of the lymphatic glands, of a deep-black colour; of an opake, somewhat humid, and homogenous tissue, having an appearance very similar to that of the bronchial glands. When this tissue begins to undergo the mollification common to productions of this class, it gives out, on pressure, a thin reddish liquid, mingled with small portions of blackish grumous matter, which are sometimes very firm, at others friable; but, even when they are friable, they present a somewhat flexible character to the touch. At a more advanced epoch of the state of softening, these grumous portions, and soon all the rest of the mass, of which they constitute part, become quite friable, and are at length reduced to a black pulp.

Melanoses may exist in four different forms: 1, that of masses enclosed in cysts; 2, that of non-encysted masses; 3, that of matter infiltrated in the tissue of some organ; 4, that of matter deposited on the surface of an organ.

These melanoses, like all accidental formations which have no analogous structure in the animal economy, produce both local and general effects. The most constant of the latter is, M. Laennec says, a gradual diminution of the vital powers, and a very remarkable alteration in the nutrition of the body. We are not quite confident of the correctness of M. Laennec's views in this instance, in regarding the derangement of nutrition expressly as an effect of the existence of the morbid productions. May not the latter, on the contrary, be an effect of the former? He says, that the patients who died in consequence of this affection, in whatever organ it was situate, and those even in which the morbid matter occupied a large portion of the lungs, had no continued or well-marked fever.

The black matter formed in this disease, is considered by M. Laennec to be essentially different in its origin from that naturally existing in the bronchial glands in the adult. The development of the former, even to a great extent, never gives rise to expectoration of black matter, excepting by chance, at the time when the melanoses become softened, and are evacuated into the cavity of the bronchiæ.

The local symptoms produced by the foregoing species of tumors are nearly similar, and consist principally in dyspnœa proportionate to their size and extent, and a cough, which is sometimes dry, and sometimes accompanied with expectoration of matter of various kinds. Sometimes, especially the *encephaloides*, produce death by suffocation, when they are very extensive; but patients of those maladies more commonly die in a state of cachectic emaciation, sometimes with, and sometimes without, hectic fever.

When the tumors are isolated and small in extent, neither percussion nor auscultation by means of the stethoscope disclose their existence; because the surrounding tissue of the lungs is permeable to the air, and in the healthy state. But, when they have acquired a considerable volume, there will of course be absence of the sound of respiration in the corresponding space. Respiration is heard perfectly in the points of the chest that correspond to ulcerated cavities, which are found after the softening of the tumors, and is more sonorous in proportion as the excavation has more extent. It seems, indeed, that the sound produced by the penetration of air into the ulcerated cavities is greater than that of respiration in a healthy lung; but the murmur and crepitation heard in the latter case, are not perceived in the former. The sound of the air rushing into these cavities is more like that of a blast of wind; and this sound, by showing that the air passes into cavities greater than the natural air-cells, may alone give rise to a presumption of their existence. When percussion produces a dull sound in any part of the chest, and respiration is heard more loudly than natural, and without the murmur or crepitation made by healthy lungs, in a portion of small extent in the part furnishing this dull sound on percussion, we may be assured that there exists in this part an ulcerous cavity situate in the middle of a portion of the lungs hardened by the close-set accumulation of tubercles. If, in this case, pectoriloquism is not developed at the moment when the examination is made, we may be assured that it will be manifested at another moment, when the temporary obstruction of the bronchiæ by the matter expectorated, or of the openings into the cavity, is removed. Several of the other phenomena of this kind, designated as appearing in phthisis, will, it must be obvious, be also observed in the cases under consideration.

*Pleurisy* is the next subject we have to treat of. M. Laennec says, that, in many cases, inflammation of the pleura and peripneumony exist simultaneously; that in cases where the pleura alone is inflamed, the pain in the side is hardly remarkable, and only at certain instants; that sometimes it is not manifest at any period of the disease; that, in other cases, on the contrary, where there exist at the same time severe peripneumony and very slight pleurisy of but little extent, there may be the most violent pain in the side. It is certain, however, that inflammation of the pleura may exist without that of the lungs, and *vice versâ*. There are constitutional epidemics in which they are commonly found isolated, and others where they are ordinarily united. In general, we more frequently find, on opening dead bodies, peripneumony without pleurisy, than pleurisy without peripneumony; because almost all cases of simple pleurisy terminate in a favourable manner.

Pleurisy may be either acute or chronic. The anatomical characters of the former are, more or less considerable redness of the pleura, in a spotted manner, leaving intervals in which the white colour of it may be discerned; enlargement of the vessels running over its surface. M. Laennec has never found a thickening of the membrane very well marked; and he thinks, that many who have thought they have observed such a change in its structure, have mistaken for it miliary tubercles developed on its surface, cartilaginous incrustations situate between this membrane and the parts covered by it, and false membranes adherent to its internal surface. Its common consequences are, serous or sero-purulent effusion; false membranes of various characters, converted into serous and cellular tissue. In chronic pleurisy, the redness is more pronounced than in the acute; the serous effusion is more abundant, less limpid, more puriform, and mingled with a great quantity of albuminous flakes, small portions of detached membraniform productions; purulent collections of various kinds, produced by the pleura and the lungs, either simply suppurated or affected with tubercles.

An acute pleurisy, of which the symptoms are very precisely marked, is ordinarily easily recognizable. The pain in the side; the dyspnœa; the fever; the cough, either dry or accompanied only by expectoration of glairy and almost colourless mucus, are often sufficient to furnish moral proofs of its existence, without having recourse to any other method of exploration; but these symptoms exist sometimes without pleurisy, and it is not rare for this affection to exist, even in the acute form, with the absence of some of those signs ; and in chronic pleurisy especially, they are often so slightly characterized, and accompanied with so many different anomalies in many of the functions, that it is not until several weeks, or even months, have elapsed, that the real nature of the disease is discovered.

Percussion will furnish more certain indications of pleurisy. As soon as collection of fluid is effected in the chest, it ceases to produce resonance in the part where the fluid is present.

Mediate auscultation furnishes even more certain means for discovering the existence of the fluid effused, and it will even indicate its extent. A great diminution, or the total absence, of the sound of respiration ; the appearance, disappearance, and return, of egophony, are the signs here manifested by means of the stethoscope. When, as it often happens, the pleuritic effusion is very abundant on its first formation, the absence of respiration is then total, uniform, and so complete, that no sound whatever can be heard, however forcibly the respiratory efforts may elevate the thoracic parietes. The only part in which there is an exception to this, is in an extent of about three fingers' breadth from the vertebral column, and this exception is constant in simple pleurisy, and may serve to distinguish this from the absence of the sound of respiration arising from inflammation of the lungs. This phenomenon appears to depend on the lungs being in contact with the parietes of the chest in the part just mentioned ; and therefore the interposition of fluid between those parts, with its pressure on the lungs, which are the cause of the absence of the sound of respiration, do not here exist. In cases where the lungs are attached to the sides of the chest, in other points, from adhesions of ancient date, the same exception may however exist in the parts thus affected. Sometimes, although the quantity of fluid effused is not diminished, the sound of respiration will return in a slight degree at the end of a few days ; but it is without any mixture with rattles, unless pulmonary catarrh exists at the same time, which may help to make its distinction from pneumonia. When the effusion is not considerable, the sound of respiration ordinarily becomes like what it is in children. When the fluid is disappearing, the sound of respiration returns from above downwards, as the surface of the fluid descends ; except in cases where this order is interrupted by adhesions of the lungs to the thoracic parietes.

To the above signs must be joined *egophony*, which is characteristic of it, and constantly indicates a collection of fluid when it is moderate in quantity ; but it disappears, when the quantity of fluid is very considerable. It is most evident where the fluid has least consistence, and therefore is generally most distinct in the situation of the upper part of the collection. The egophony descends in the zones the fluid abandons in succession when this is removed by absorption, and the sound of respiration returns in the parts as the egophony disappears. Egophony may be suspended during some minutes, or even hours : it re-appears when the patient expectorates.

Many authors have noticed an increase in the volume of the side in which the collection of fluid exists, especially in chronic pleurisy. M. Laennec states, that he has several times seen this exist in a very remarkable manner: it disappears as the cure advances; and the side occasionally becomes, at length, smaller than in the natural state.

This leads us to some observations on a contraction of the side, after some cases of pleurisy, that appear to us to be extremely interesting. These cases, less rare than might be supposed, says M. Laennec, is one of those that has never been well described. The subjects in whom it exists are recognizable by their exterior conformation, and their position when erect. They seem to rest expressly on the affected side, even when they endeavour to stand erect. The chest is narrower on this than on the healthy side, and, if it be measured, there will often be found as much as an inch difference between them: its extent in length is equally diminished; the ribs are more closely approached to each other; the muscles, and particularly the pectoralis major, have not above half the volume of those of the opposite side. This contraction depends on a peculiar mode of termination of chronic pleurisy, or of acute pleurisy become chronic. The sero-purulent effusion having continued for a long time, the false membranes which cover the pleura of the lungs, as well as that lining the ribs, acquire a peculiar sort of firmness, and a degree of organization which renders them insusceptible of being converted into cellular tissue. When the collected fluid is at length absorbed, the lung compressed for so long a time, and maintained also in that state by a thick strong false membrane which envelopes it throughout, cannot become dilated with sufficient promptness to permit it to follow the progress of the removal of the effused fluid: the ribs then approach each other, and the chest is contracted in volume. When the fluid is totally absorbed, the costal and pulmonary portions of the false membrane come in contact, and soon become adherent in so intimate a manner, that it might be believed they formed only one and the same membrane, the texture of which becomes more and more firm, and terminates, at the end of some months, by acquiring the consistence and all the characters of a fibrous or fibro-cartilaginous membrane.

Sometimes fibro-cartilaginous membranes are found in pleurisy, in some few instances unaccompanied with any considerable degree of serous or purulent effusion. In this form of pleurisy, the pain in the side is rare, fugacious, and often so slight, that patients do not complain of it, at least not unless they are interrogated respecting it. The difficulty of respiration is sometimes but little remarkable; the cough is seldom, and dry. The pectoral symptoms are frequently those least attended to. A state of languor and extreme weakness, a febrile disturbance but little marked by the pulse, a disproportionate degree of anorexy, in respect to the duration and apparent gravity of the malady, are often the only symptoms which openly present themselves.

The stethoscope and percussion are the only means for recognizing the nature of the disease. Percussion alone could, however, only give rise to a suspicion of its existence: we could not, by this mean, become assured whether the absence of sound depended on a state of engorgement of the lung, or a pleuritic effusion ; it would not indicate any thing, if the seat of the disease be confined to the inferior part of the right side of the chest. But, in joining with it mediate ausculta-tion, the absence of respiration every-where else but about the roots of the lungs, leaves no doubt respecting the existence of the effusion.

*Gangrene of the pleura*, is of very rare occurrence: it is hardly ever general, or even extensive. M. Laennec has never seen a case in which it appeared to have been an effect of the violence of acute inflammation. Most frequently, it only takes place in consequence of a bursting into the cavity of the pleura of a gangrenous abscess of the lungs. Sometimes it happens in chronic inflammation, when the disease has been of a certain duration.

*Dropsies of the pleura*, are distinguished into two species by M. Laennec: idiopathic, or that unattended with apparent disease of some of the solid contents of the chest; and symptomatic, or that which arises from some apparent organic lesion. The first he considers to be a very rare disease: that is, it is very rare that it exists alone to such an extent as to cause death ; although it is commonly believed to be of very frequent occurrence. This error has arisen from the *serous* or *sero-purulent* exhalation formed in chronic pleurisy being mistaken for it. He does not think that the proportion is greater than one in two thousand bodies. It ordinarily exists only on one side. When the collection of fluid is very considerable, the affected side is rendered more voluminous than the other.

The principal and almost only local symptom of this malady, is difficulty of respiration. Percussion gives rise to a dull sound ; and the stethoscope renders manifest the sound of respiration only about the root of the lung. Egophony may also be sometimes joined with those signs.

The general symptoms and the progress of the disease may, in some instances, be the only means of enabling us to distinguish it from chronic pleurisy.

Hydrothorax from disease of the pleura, or some other of the thoracic organs, is as common an affection as the foregoing one is rare. It is most frequently met with in the bodies of persons dying of acute fevers, diseases of the heart, or tubercles of some of the thoracic organs. Its signs, similar in every respect to those of idiopathic hydrothorax, ordinarily do not begin to develop themselves until a few days, or even a few hours, before death. Sometimes a considerable quantity of water is found in both sides of the chest of persons who had not experienced any remarkable dyspnœa. M. Laennec says, "May we not think that, in this case, the effusion did not happen until the moment of death, or in the first instants following it."

*Effusion of blood into the cavity of the pleura*, may arise from injuries or diseases of the thoracic organs, *pulmonary apoplexy*, or spontaneous hemorrhage from the pleura. Percussion and mediate auscultation furnish the same signs as in the effusion from pleurisy.

*Pneumo-thorax*, is a collection of aëriform fluids in the cavity of the pleura. In some instances this fluid is inodorous, but more frequently fetid, exhaling an odour analogous to that of sulphurated hydrogen.

The quantity is sometimes so great as to press the lung almost close to the vertebræ, and distend the chest in a very sensible manner. The ribs are separated; the diaphragm, thrust towards the abdominal cavity, forms a considerable projection in it, when the aëriform collection is on the left side; if it be on the right, the liver is driven down below the false ribs.

Although this malady is not extremely rare, it has but little engaged the attention of physicians. M. ITARD, who wrote a dissertation on it, regarded it as an affection consecutive to phthisis, and produced by the decomposition of stagnant pus in the chest. M. Laennec admits two other species: one, from a communication of the bronchial canals with the cavity of the pleura; the other, from a sort of gaseous exhalation. The probability of the latter seems to be supported by some observations of Sir EVERARD HOME and Dr. WILMOT on exhalations of this sort. It may arise, too, from a gangrenous state of the lungs.

The symptoms of pneumo-thorax are but little known, and are very obscure; as those detected are common to some other affections. The only one which is constant, is some degree of dyspnœa. Percussion alone furnishes no decisive evidence. When the collection is very considerable, the affected side renders a more clear sound than the healthy one. But this difference, instead of favouring the discovery of the disease, tends rather to produce further error, by making us suspect that the healthy side is suffering engorgement or some other malady. But the true sign of this affection may be found by a comparison of the results obtained by mediate auscultation with those arising from percussion. When, in a person whose chest sounds better on one side than on the other, respiration is well heard in the side the least sonorous, whilst it is not perceived at all in the other, we may be assured that pneumo-thorax exists in the latter side. The same diagnostic might be established, even when the two sides of the chest may be equally sonorous.

One circumstance may, in some cases, render the diagnosis difficult; that is, when the lung adheres to a certain extent to the costal pleura, by short bands of cellular tissue. Here respiration may be heard in the side affected with pneumo-thorax. It may not be easy, in some cases, to distinguish it from emphysema of the lungs. The history of the affection will however, in most cases, much facilitate the diagnosis.

When pneumo-thorax exists with a collection of liquid fluid, this case may be distinguished by the complete absence of the sound from respiration and percussion in the parts occupied by the liquid, and the absence of respiration only in those occupied by the gas. The *metallic ringing* was also discovered in one case of this kind. The latter phenomenon is the only sign by which we can recognize the communication of the pleura with the canals of the bronchiæ, in the case of empyema joined with pneumo-thorax. The discovery of this is of much importance in practice; because the success of the operation of paracentesis of the thorax would be very doubtful, though it is not impossible under these circumstances; as the cases related by Bacqua,

James, and Robins, where the patients recovered after this operation, although part of the injections they made into the cavity of the pleura passed into the mouth. On the other hand, the state of simple pneumo-thorax is apparently, as HEWSON and RULLIER remarked, the case in which paracentesis might be performed with the greatest prospect of a favourable issue.*

---

*Researches respecting the Situation of the internal Orifice of Fistula in the Anus, and on the Parts which the Ulcers traverse in this Disease.* By M. RIBES, M.D.

THE results of these researches of M. Ribes possess a considerable degree of interest, from the influence they must have on practical means, should his remarks be applicable to the generality of cases; and that they are so, we have but little doubt, from the situation of the internal orifice of this fistula having been such as he describes in nearly every instance that has occurred to our own observation. The author connects his original views with a general account of the anatomy of the parts about the anus; but we shall confine our abstract to the former part of his memoir, adding such points of the latter as are necessary to render our description perspicuous.

The internal sphincter muscle and the hemorrhoidal plexus are situate at the inferior part of the rectum, between the mucous tunic and the longitudinal fleshy fibres of the intestine. Interiorly, the hemorrhoidal plexus is in contact with the internal membrane of the rectum, and when any of the branches of this plexus become dilated in this point, the above-mentioned membrane is distended inwardly, it becomes thin, and assumes a bluish colour, so that it seems as if the tumors projected into the intestinal canal without the interposition of this membrane, which appears no longer to exist. But, if these tumors be carefully dissected, the mucous tunic of the rectum may be removed, and we then discover the proper membrane of the hemorrhoidal tumor. Externally, the hemorrhoidal plexus is situate on the internal sphincter muscle, and it is of much importance that it should be understood, that very large branches are here given off from this plexus, traverse the muscle, pass outwardly, descend on the external surface of this muscle as far as its lower margin, and then communicate again with the inferior portion of the hemorrhoidal plexus; so that the internal sphincter muscle, in persons who are severely affected with hemorrhoids, is traversed, and as it were embraced, by several large veins, which give it a sort of cavernous aspect.

M. Ribes found, that air blowed into the inferior mesenteric veins distended the hemorrhoidal plexus, and rendered the cellular tissue about the lower part of the rectum emphysematous. Coloured injections passed in the same way.

---

* We shall conclude the analysis of this work in the next Number. It is not possible to give any thing like an adequate idea of its contents, without entering into the details in which we have indulged.

The hemorrhoidal veins, when morbidly distended with blood, form simple varices; but if, from any cause, the blood, instead of flowing upwards through the veins, descends and becomes effused into the cellular tissue about the anus, with which those vessels communicate, a sort of pouch is formed, which projects into the canal of the rectum, and constitutes the proper hemorrhoid. If the inferior mesenteric vein be traced in a subject affected with hemorrhoids, the ramifications of that vessel will be found to terminate in the sanguineous pouches above described. It should be well understood, that these pouches are found in the cellular tissue, and are distinctly extraneous to the veins. If the parts in question be well dissected and completely removed, the hemorrhoids will remain suspended to the branches of the hemorrhoidal vein, like a bunch of grapes on its stalk.

These tumors are often affected with pain and inflammation. When they rupture externally, blood is evacuated; and the patient is relieved until the return of a new attack. But, when the hemorrhoids are situate internally and penetrate the canal of the rectum, the blood is poured into this intestine, and evacuated by the anus. Now, in this case, the fluid part of the fæces may filtrate through the ulcerated point, penetrate the hemorrhoidal pouch, produce inflammation and suppuration in it, and, consequently, give rise to a fistula.

In a hundred cases of fistula in the anus, ninety-nine, M. Ribes states, are formed in the way above described. Hence, the internal orifice of the fistula is most frequently found immediately above the part where the internal membrane of the rectum unites with the skin, and sometimes a little higher; but, in seventy-five subjects, examined by the author, the opening was never more than five or six lines higher up than the point just mentioned, and in several the distance was not above three or four lines.

The course in which the fistulas ran was various. In several cases, after taking its origin from the interior of the rectum, it descended between the mucous membrane of this intestine and the internal sphincter muscle, and, having arrived at the lower part of this, it turned outwards, ran between the external sphincter and the skin, and then opened in the neighbourhood of the margin of the anus. In several other cases, it passed through the fibres of the internal sphincter, then descended between this muscle and the longitudinal fleshy fibres of the rectum, ran to the superior surface of the external sphincter, traversed the fibres of this muscle, and then ended in an ulceration of the skin near the inferior extremity of the rectum. From these observations M. Ribes is convinced that, in almost all cases, the fistula is developed in an hemorrhoid, and sometimes in the course of one of the veins of the hemorrhoidal plexus.

In the greater number of cases, then, the internal orifice of fistulas may be discovered externally, if the patient be desired to make an effort to evacuate the rectum; or, if it be not readily discerned by the eye, it may be discovered by means of a probe. In order to arrive at it by the external opening, the probe should be passed horizontally, and almost closely parallel to the perineum. The difficulty often experienced in finding it, arises from the instrument being passed too high upwards: it should be slided along the internal surface of the skin without abandoning it, and then directed to the inferior part of the rectum: in this way we are sure soon to find the internal opening and the finger which had been previously passed into the rectum. If the patient be conveniently disposed, and we wish to operate by incision, the extremity of the instrument may be passed out at the extremity of the anus by bending it a little, and a bistoury passed along it from the external opening towards the anus, until the integuments are divided from one extremity of the fistula to the other. M. Ribes uses, we believe, a flexible grooved sound instead of a simple probe, when he traces the fistula as above described; that being better to cut upon than a probe, and, by using it in the first instance, the necessity of passing an instrument twice is avoided.

M. Ribes considers that we should never operate when the probe does not penetrate into the cavity of the rectum by the internal orifice of the fistula, and that we need not feel any anxiety about the denudation of the intestine when the two openings of the fistula are comprised in the incision. In the case of merely external, or, as it is sometimes termed, blind fistula, the denudation of the rectum is never sufficiently great, nor the cellular tissue so extensively destroyed, as to lead us to despair of the union of the intestine with the adjacent parts; and, according to Sabatier, the intestine should never be divided in this instance.

From the preceding account, we should be led to consider that fistula of the anus is ordinarily a disease of but little importance; at least, that the proper operation for it, in the generality of cases, is one of the most simple in surgery.

---

